# The Distribution, Diversity, and Control of Dirofilariosis in Brazil: A Comprehensive Review

**DOI:** 10.3390/ani14172462

**Published:** 2024-08-24

**Authors:** Marianna Laura Elis Chocobar, Elizabeth Moreira dos Santos Schmidt, William Weir, Rossella Panarese

**Affiliations:** 1 School of Veterinary Medicine and Animal Science (FMVZ), São Paulo State University (UNESP), Rua Prof. Dr. Walter Maurício Corrêa, s/n, Botucatu 18618-681, SP, Brazil; laura.elis@unesp.br (M.L.E.C.); elizabeth.schmidt@unesp.br (E.M.d.S.S.); 2 School of Biodiversity, One Health and Veterinary Medicine, College of Medical, Veterinary and Life Sciences, University of Glasgow, Garscube Estate, Glasgow G61 1QH, UK; willie.weir@glasgow.ac.uk

**Keywords:** *Dirofilaria immitis*, *Dirofilaria repens*, mosquito-borne disease, One Health, diagnosis, genomics

## Abstract

**Simple Summary:**

Dirofilariosis, a zoonotic mosquito-borne disease, remains an under-studied condition in several areas of Brazil, despite its long-standing presence. This review aims to draw together prevalence data from epidemiological studies and case reports conducted in dogs over the last decade and in cats, wildlife, and humans over the last twenty years to define the distribution and prevalence of *Dirofilaria* spp. within the country and highlight the challenges associated with its diagnosis, treatment, and control. While *Dirofilaria immitis* is the main species circulating in Brazil, concerns have been raised in recent years about the presence of genetically distinct strains that may be more virulent than their Old World counterparts. In light of this and the widespread distribution of the parasite among both Brazilian coastal and non-coastal areas, further studies are required to characterise the disease. Additionally, there is a need to increase awareness of this neglected disease among health professionals in Brazil and to understand and minimise its impact on both veterinary and public health.

**Abstract:**

Brazil’s extensive coastline, tropical and subtropical climate, and well-preserved environment represent a conducive setting for dirofilariosis, a zoonotic mosquito-borne disease. Although this condition has long been recognised in the country, it has been relatively under-studied, and it is currently considered to be an emerging disease. Diagnosis, treatment, and control remain challenging due to the extensive gaps in knowledge. In order to help address this issue, this review aims to (i) summarise the available literature on the distribution of *Dirofilaria* spp. in Brazilian dogs over the last decade, (ii) review case reports of dirofilariosis in cats, wild animals, and humans over the last twenty years, and (iii) highlight the benefits of taking a One Health approach to managing this disease. While there have been several prevalence studies in dogs, disease distribution is poorly characterised in cats, and little is known about the occurrence of the parasite in wildlife. Human cases are sporadically reported, and no large-scale studies have been undertaken to date. Evidence indicates that *Dirofilaria immitis* is the main species circulating in Brazil, although *Dirofilaria repens* has also been detected. Molecular studies have also suggested the circulation of a highly virulent form of *D. immitis*, which may be genetically distinct from those of the Old World. A programme of epidemiological, ecological, genomic, and pathogenicity-based studies is required to quantify the impact of dirofilariosis in Brazil on both veterinary and public health and to inform others on its control.

## 1. Introduction

Dirofilariosis is a zoonotic mosquito-borne disease that can affect a variety of mammalian species, including dogs, cats, and humans, and is caused by filarial nematodes belonging to the *Dirofilaria* genus. Among these, *Dirofilaria immitis* and *Dirofilaria repens* are considered the most important species due to their impact on both veterinary and public health. *Dirofilaria repens* is widely distributed in dogs and humans in Europe and has also been described in the Middle and Far East [1,2,3], Africa [4], Brazil [5], Chile [6], and, recently, Colombia [7]. *Dirofilaria immitis* is present throughout the world, being found in tropical, sub-tropical, and temperate zones, and is the most common species affecting dogs, cats, wild animals, and humans in the Americas [8,9,10].

First described by Leidy in 1856, *D. immitis* is responsible for canine Heartworm Disease (HWD) and Heartworm-Associated Respiratory Syndrome (HARD) in cats, while *D. repens*, described by Railliet and Henry in 1911, is the causative agent of Subcutaneous Dirofilariosis (SCD) [1,11,12]. In animals, *D. immitis* causes severe disease that may be fatal if not properly treated, while, in contrast, *D. repens* often results in asymptomatic infections [8,13]. Both species are zoonotic, giving rise to different clinical presentations in humans. These accidental hosts can be infected by *D. repens* and *D. immitis*, which cause Human Pulmonary Dirofilariosis (HPD) or subcutaneous/intraocular dirofilariosis, respectively, along with an intense inflammatory response [8,13]. Dogs play an important role in the epidemiology of dirofilariosis by acting as reservoirs of the infection and, due to their proximity to humans, facilitate the zoonotic transmission cycle [13], although they do not directly transmit the parasite to man. Mosquitoes are necessary for the completion of the *Dirofilaria* spp. life cycle and for enabling its survival in the environment [8].

Brazil’s ecosystem, characterised by extensive coastal borders and tropical and subtropical climates, has facilitated the establishment, perpetuation, and spread of *Dirofilaria* spp. [14,15], alongside the proliferation of its main mosquito-vector species, including *Ochlerotatus* spp., *Culex* spp., *Anopheles* spp., and *Aedes* spp. [12,16,17]. The first report of dirofilariosis due to *D. immitis* was recorded by Silva-Araújo during surveillance of the canine population in 1878 [18]. Nine years later, Magalhães described the disease in a child from Rio de Janeiro after finding a filariid in the patient’s heart and named it *Dirofilaria magalhensi* [19]. To the best of our knowledge, the first report in cats was made just prior to 1920, when single specimens were found in two cats’ hearts during necropsy [20]. While *D. immitis* has been widely reported among dogs, cats, humans, and wildlife from Brazil, *D. repens* has only been described twice in the country. On the first occasion, in 1918, it was reported in a dog from São Paulo [21]; the second time, one hundred years later in 2017, it was detected in a ring-tailed coati (*Nasua nasua*) in southern Brazil at Parque Nacional do Iguaçu, Paraná State [5]. Presently, *D. repens* is still considered non-endemic in the country.

Although several prevalence studies have investigated the distribution of *Dirofilaria* spp. in Brazil [9], these have focused mainly on *D. immitis* detection, and the methodology used has been described as limited [22,23]. Additionally, there has been a presumed under-reporting of cases of human dirofilariosis, which has been attributed to misdiagnosis [24]. Together these factors call into question the reliability of currently available data on the epidemiology of this filarial nematode in the country. Therefore, this review aims to (i) summarise the available literature on the distribution of *Dirofilaria* spp. in Brazilian dogs over the last decade, (ii) review case reports of dirofilariosis in cats, wild animals, and humans over the last twenty years, and (iii) highlight the benefits of taking a One Health approach to managing this disease.

This review draws together the results of all parasitological, serological, and molecular studies on *Dirofilaria* spp. in Brazil performed in dog populations over the last ten years and in cats, wild animals, and humans since 2004. This information was used to construct a knowledge base representing the current understanding of parasite distribution and prevalence across the country. It is presented here in terms of percentage positivity among the canine, feline, and wildlife populations surveyed and is subdivided by region, city, and host species in order to illustrate trends and highlight gaps in knowledge. For both clarity and completeness, the diagnostic method used for each study is presented, allowing all appropriate caveats to be applied in the interpretation of the data.

## 2. Distribution of *Dirofilaria* spp. in Brazil

### 2.1. Canine Hosts

**Figure 1:**
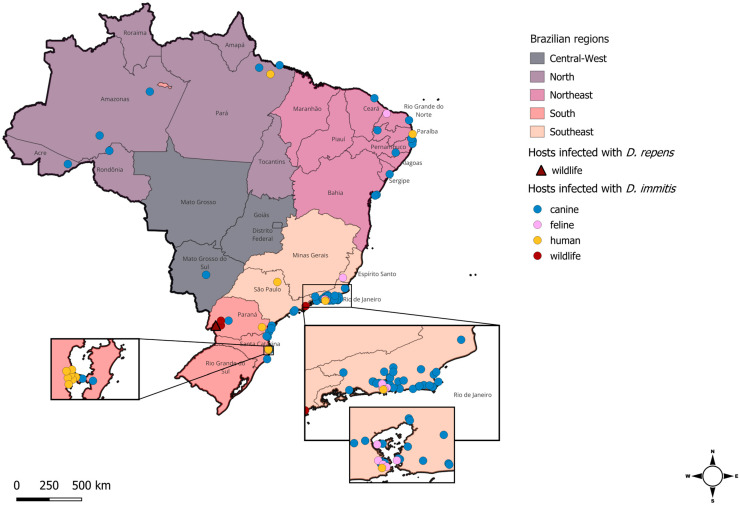
Geographical distribution of *Dirofilaria* spp. in Brazil showing cases identified in canine hosts over the last decade (2014–2024), and in feline, wildlife, and human hosts over the past twenty years (2004–2024). The locations of the dots on the map indicate the municipalities where each study was conducted, while the colour defines the host type found to be infected with either filarial species. Note: *Dirofilaria repens* infection, indicated on the map with the triangle, has been detected only once in a wildlife host, a ring-tailed coati.

Although data regarding the occurrence of *D. immitis* in the canine population have been obtained from all five Brazilian regions over the past ten years, prevalence studies have been undertaken almost exclusively in coastal areas, and little is known about the presence of dirofilariosis in the interior of the territories. Table 1 contains a comprehensive list of all the reports and prevalence studies in Brazilian dogs over the last decade alongside information regarding locality, sample size, and diagnostic method employed; these findings are summarised graphically in Figure 1. Among these studies, a certain degree of variation among prevalence levels can be observed, including intra-regional differences, and this may be explained at least in part by the diagnostic method employed, the area of sampling (i.e., rural, city, coastal, or mountainous), or whether the dogs were owned or stray [25,26,27]. For instance, the northern region, Lábrea, Amazonas State, was found to have the highest prevalence of *D. immitis* infection (44.4%) after the molecular testing of 99 dogs, most of which were from rural areas [25]. Meanwhile, researchers found a very low prevalence in the historically endemic region of Manaus, Amazon State, with only 3.7% positivity among the 766 rural canine samples analysed using only a thick blood smear [27].

In the northeast region, the lowest prevalence of *D. immitis* in dogs was found in Fortaleza, Ceará State, with only 1.1% of dogs being microfilaraemic [28]. Although the sample size was large (2400 dogs), the diagnostic technique utilising blood smear evaluation might have led to a high proportion of false-negative results. A similar prevalence was observed in Aracaju, Sergipe, where 3.7% of 378 dogs tested positive using the modified Knott’s method [29]. In contrast, in Bahia State, 20% of dogs were found to be seropositive in the cities of Salvador and Lauro de Freitas [30]. The authors commented that the prevalence in Salvador was much higher than the 5.4% previously recorded in 1991, while the current prevalence in Lauro de Freitas remained comparable to the 23.3% recorded in 1991 [30]. The highest prevalence in the region was instead found in Itamaracá, Pernambuco State [30], with almost half of the 109 samples being seropositive (49.5%), a much higher prevalence than the 29% previously reported in 1987. These findings underscore the need for frequent epidemiological surveys in order to accurately assess parasite prevalence in the canine population, as it may vary considerably over time.

**Figure 2:**
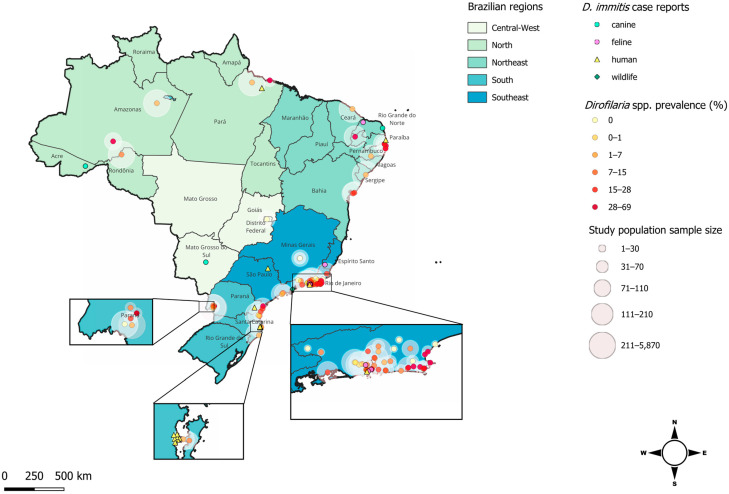
Prevalence of *Dirofilaria immitis* and *Dirofilaria repens* infections across Brazil evaluated over the last ten years (2014–2024) in dogs and twenty years (2004–2024) in cats, wildlife, and humans. To appreciate the scale of the various epidemiological studies, the sample size is indicated, together with the location of individual case reports.

In the southeast region, Rio de Janeiro is the state with the highest number of prevalence studies, encompassing twelve municipalities (Table 1; Figure 1 and Figure 2). In a study performed in Paraíba State (northeastern Brazil), a group of researchers compared different diagnostic methods in Cabo Frio, State of Rio de Janeiro. In this study, substantial differences in prevalence were estimated using each technique, with the lowest prevalence recorded by PCR (15.5%) and the highest prevalence (29.1%) recorded by the commercially available antigen detection test (SNAP 4Dx Plus Test^®^, IDEXX Laboratories, Westbrook, ME, USA), while the prevalence recorded by the modified Knott’s test was 19.4% [22]. The highest prevalence recorded in the last ten years was in Araruama, a sandy area in the state of Rio de Janeiro, where both Knott’s and commercial antigen tests were performed (68.9%) [31].

In the southern region, the highest prevalence was found in Guaraqueçaba, Paraná (31.8%) [30]. The presence of the parasite was confirmed in a non-coastal area in Parque Nacional Iguaçu, a well-conserved Atlantic Forest area located in Paraná State. Here, the *D. immitis* prevalence in domestic dogs was found to be 16% by the modified Knott’s test and 22% using a commercial antigen detection test [5].

Finally, the central-west region is the least studied area in Brazil for *Dirofilaria* spp. prevalence. Only one location was investigated, revealing a prevalence of 7.1% [32], and just a single case of disease was reported [33]. This result provides evidence of the presence of *Dirofilaria* spp. in the southwestern part of Brazil and serves to encourage further research in non-coastal areas.

**Table 1:** *Dirofilaria immitis* prevalence studies conducted in dogs in Brazil between 2014 and 2024, classified by region, state, city, and diagnostic tests employed.

Region	State	City	Filarial Species	Prevalence	Positives/Total	Diagnostic Method	Authors
North	AM	Lábrea	*D. immitis*	44.4%	44/99	PCR (cox1)	[25]
	AM	Manaus	*D. immitis*	3.7%	28/766	Thick blood smear	[27]
	PA	Ilha do Algodoal	*D. immitis*	35.8%	24/67	Knott’s method and PCR (12S)	[26]
	PA	Ilha de Marajó	*D. immitis*	2.1%	9/418	PCR (5.8S-ITS2-28S)	[34]
	RO	Porto Velho	*D. immitis*	12.8%	93/727	Immunochromatography (CHW Ag 2.0 Test Kit, Alere Bionote Inc., Gyeonggi-do, Republic of Korea)	[35]
	AC	Rio Branco	*D. immitis*	Case Report	1/1	Clinical examination, Microscopy, SNAP 4Dx Plus Test (IDEXX Laboratories), Echocardiogram	[36]
Northeast	BA	Lauro de Freitas	*D. immitis*	20.3%	30/148	Serology (Witness Dirofilaria, Zoetis®, Parsippany, NJ, USA)	[30]
	BA	Salvador	*D. immitis*	20.0%	24/120	Serology (Witness Dirofilaria, Zoetis®, Parsippany, NJ, USA)	[30]
	CE	Fortaleza	*D. immitis*	1.1%	26/2400	Blood smear	[28]
	PB	Sousa	*D. immitis*	33.6%	48/140	Knott’s method, Immunochromatography (CHW Ag 2.0 Test Kit, Alere Bionote Inc., Republic of Korea), and PCR (COI)	[37]
	PE	Recife	*D. immitis*	36.7%	22/60	Serology (Witness Dirofilaria, Zoetis®, Parsippany, NJ, USA)	[30]
	PE	Itamaracá	*D. immitis*	49.5%	54/109	Serology (Witness Dirofilaria, Zoetis®, Parsippany, NJ, USA)	[30]
	PE	Recife	*D. immitis*	11.5%	12/104	Knott’s method	[38]
	PE	Goiana	*D. immitis*	36.3%	74/204	SNAP 4Dx Plus Test (IDEXX Laboratories)	[39]
	PE	São Joaquim de Bicas	*D. immitis*	0.0%	0/103	SNAP 4Dx Plus Test (IDEXX Laboratories)	[39]
	PE	Garanhuns	*D. immitis*	5.5%	11/201	SNAP 4Dx Plus Test (IDEXX Laboratories)	[40]
	PE	Goiana	*D. immitis*	32.0%	32/100	SNAP 4Dx Plus Test (IDEXX Laboratories)	[41]
	SE	Aracaju	*D. immitis*	3.7%	14/378	Knott’s method	[29]
	RN	Natal	*D. immitis*	Case report	1/1	Skin cytology, histopathology, Knott’s method, Thick smear, PCR	[42]
Southeast	RJ	Cabo Frio	*D. immitis*	30.1%	31/103	Microscopy, SNAP 4Dx Plus Test (IDEXX Laboratories), and PCR (COI)	[22]
	RJ	Mangaratiba	*D. immitis*	16.3%	23/141	Serology (Witness Dirofilaria, Zoetis®, Parsippany, NJ, USA)	[30]
	RJ	Niterói	*D. immitis*	58.6%	92/157	Serology (Witness Dirofilaria, Zoetis®, Parsippany, NJ, USA)	[30]
	RJ	Cabo Frio	*D. immitis*	27.5%	11/40	Serology (Witness Dirofilaria, Zoetis®, Parsippany, NJ, USA)	[30]
	RJ	Armação de Búzios	*D. immitis*	62.2%	23/37	Serology (Witness Dirofilaria, Zoetis®, Parsippany, NJ, USA)	[30]
	RJ	Rio de Janeiro	*D. immitis*	7.0%	30/428	SNAP 4Dx Plus Test (IDEXX Laboratories)	[23]
	RJ	Rio de Janeiro	*D. immitis*	21.6%	44/204	Microfilariae and antigen detection	[43]
	RJ	Rio de Janeiro	*D. immitis*	5.8%	6/103	Immunochromatography (CHW Ag 2.0 Test Kit, Alere Bionote Inc., Gyeonggi-do, Republic of Korea)	[44]
	RJ	Araruama	*D. immitis*	25.0%	1/4	Direct examination and SNAP 4Dx Plus Test (IDEXX Laboratories)	[45]
	RJ	Barra de São João	*D. immitis*	27.8%	5/18	Direct examination and SNAP 4Dx Plus Test (IDEXX Laboratories)	[45]
	RJ	Cabo Frio	*D. immitis*	14.6%	12/82	Direct examination and SNAP 4Dx Plus Test (IDEXX Laboratories)	[45]
	RJ	Campos	*D. immitis*	17.5%	29/166	Direct examination and SNAP 4Dx Plus Test (IDEXX Laboratories)	[45]
	RJ	Iguaba Grande	*D. immitis*	30.0%	3/10	Direct examination and SNAP 4Dx Plus Test (IDEXX Laboratories)	[45]
	RJ	Macaé	*D. immitis*	0.0%	0/18	Direct examination and SNAP 4Dx Plus Test (IDEXX Laboratories)	[45]
	RJ	Maricá	*D. immitis*	24.8%	61/246	Direct examination and SNAP 4Dx Plus Test (IDEXX Laboratories)	[45]
	RJ	Rio das Ostras	*D. immitis*	31.5%	17/54	Direct examination and SNAP 4Dx Plus Test (IDEXX Laboratories)	[42]
	RJ	Saquarema	*D. immitis*	9.4%	3/32	Direct examination and SNAP 4Dx Plus Test (IDEXX Laboratories)	[45]
	RJ	Cachoeiras de Macacu	*D. immitis*	0.0%	0/17	Direct examination and SNAP 4Dx Plus Test (IDEXX Laboratories)	[45]
	RJ	Metropolitan Region	*D. immitis*	15.2%	14/92	Direct examination and SNAP 4Dx Plus Test (IDEXX Laboratories)	[45]
	RJ	Itaboraí	*D. immitis*	7.1%	2/28	Direct examination and SNAP 4Dx Plus Test (IDEXX Laboratories)	[45]
	RJ	Magé	*D. immitis*	27.3%	3/11	Direct examination and SNAP 4Dx Plus Test (IDEXX Laboratories)	[45]
	RJ	Niterói	*D. immitis*	21.1%	22/104	Direct examination and SNAP 4Dx Plus Test (IDEXX Laboratories)	[45]
	RJ	Nova Iguaçu	*D. immitis*	5.4%	2/37	Direct examination and SNAP 4Dx Plus Test (IDEXX Laboratories)	[45]
	RJ	Rio de Janeiro	*D. immitis*	20.0%	2/10	Direct examination and SNAP 4Dx Plus Test (IDEXX Laboratories)	[45]
	RJ	São Gonçalo	*D. immitis*	15.2%	14/92	Direct examination and SNAP 4Dx Plus Test (IDEXX Laboratories)	[45]
	RJ	Maricá	*D. immitis*	7.5%	6/80	Knott’s method	[46]
	RJ	São Pedro da Aldeia	*D. immitis*	43.4%	23/53	Knott’s method	[46]
	RJ	Maricá/Niterói	*D. immitis*	24.1%	33/137	Knott’s method	[47]
	RJ	Guapimirim	*D. immitis*	1.5%	20/1372	Immunochromatography (CHW Ag 2.0 Test Kit, Alere Bionote Inc., Gyeonggi-do, Republic of Korea), ELISA, and Knott’s method	[48]
	RJ	São João de Meriti	*D. immitis*	0.2%	1/667	Immunochromatography (CHW Ag 2.0 Test Kit, Alere Bionote Inc., Gyeonggi-do, Republic of Korea), ELISA, and Knott’s method	[48]
	RJ	Nova Iguaçu	*D. immitis*	0.9%	2/226	Immunochromatography (CHW Ag 2.0 Test Kit, Alere Bionote Inc., Gyeonggi-do, Republic of Korea), ELISA, and Knott’s method	[48]
	RJ	Magé	*D. immitis*	8.2%	156/1896	Immunochromatography (CHW Ag 2.0 Test Kit, Alere Bionote Inc., Gyeonggi-do, Republic of Korea), ELISA, and Knott’s method	[48]
	RJ	Duque de Caxias	*D. immitis*	1.8%	107/5870	Immunochromatography (CHW Ag 2.0 Test Kit, Alere Bionote Inc., Gyeonggi-do, Republic of Korea), ELISA, and Knott’s method	[48]
	RJ	Ilha do Governador	*D. immitis*	30.2%	19/63	SNAP 4Dx Plus Test (IDEXX Laboratories)	[49]
	RJ	Araruama	*D. immitis*	68.9%	115/167	Knott’s method and SNAP 4Dx Plus Test (IDEXX Laboratories)	[32]
	RJ	Aldeia Velha	*D. immitis*	7.5%	8/106	Knott’s method and SNAP 4Dx Plus Test (IDEXX Laboratories)	[32]
	RJ	São Vicente	*D. immitis*	0.0%	0/60	Knott’s method and SNAP 4Dx Plus Test (IDEXX Laboratories)	[32]
	MG	Igarapé	*D. immitis*	0.0%	0/50	SNAP 4Dx Plus Test (IDEXX Laboratories)	[42]
	MG	São Joaquim de Bicas	*D. immitis*	0.0%	0/50	SNAP 4Dx Plus Test (IDEXX Laboratories)	[42]
	MG	Uberlândia	*D. immitis*	Case Report	1/1	Urinalysis, Thick blood smear	[50]
	SP	Guarujá	*D. immitis*	2.8%	4/142	Serology (Witness Dirofilaria, Zoetis®, Parsippany, NJ, USA)	[31]
	SP	Bertioga	*D. immitis*	7.6%	7/92	Serology (Witness Dirofilaria, Zoetis®, Parsippany, NJ, USA)	[31]
South	PR	Parque Nacional Iguaçu	*D. immitis*	22%	11/50	Serology (Witness Dirofilaria, Zoetis®, Parsippany, NJ, USA)	[5]
	PR	Parque Nacional Iguaçu	*D. immitis*	0.0%	0/225	Knott’s method, Immunochromatography (CHW Ag 2.0 Test Kit, Alere Bionote Inc., Gyeonggi-do, Republic of Korea), and PCR (myoHC and hsp70)	[51]
	PR	Guaratuba	*D. immitis*	24.5%	12/49	Serology (Witness Dirofilaria, Zoetis®, Parsippany, NJ, USA)	[31]
	PR	Guaraqueçaba	*D. immitis*	31.8%	7/22	Serology (Witness Dirofilaria, Zoetis®, Parsippany, NJ, USA)	[31]
	PR	Pontal do Paraná	*D. immitis*	26.3%	31/118	Serology (Witness Dirofilaria, Zoetis®, Parsippany, NJ, USA)	[31]
	SC	Florianópolis	*D. immitis*	2.1%	3/146	Serology (Witness Dirofilaria, Zoetis®, Parsippany, NJ, USA)	[31]
	SC	Araquari	*D. immitis*	7.3%	11/150	Serology (Witness Dirofilaria, Zoetis®, Parsippany, NJ, USA)	[31]
	SC	Laguna	*D. immitis*	4.6%	11/238	Knott’s method, Immunochromatography (CHW Ag 2.0 Test Kit, Alere Bionote Inc., Gyeonggi-do, Republic of Korea), and PCR (surface antigen gene)	[52]
	SC	Joinville	*D. immitis*	0.7%	3/429	Knott’s method and SNAP 4Dx Plus Test (IDEXX Laboratories)	[53]
Central-West	MT	Pantanal—Barão de Melgaço	*D. immitis*	7.1%	6/84	SNAP 4Dx Plus Test (IDEXX Laboratories)	[33]
	DF	Brasilia	*D. immitis*	0.0%	0/100	SNAP 4Dx Plus Test (IDEXX Laboratories)	[42]
	MS	Campo Grande	*D. immitis*	Case Report	1/1	Knott’s method, SNAP 4Dx Plus Test (IDEXX Laboratories) and PCR (12S)	[34]

AM = Amazonas; PA = Pará; RO = Rondônia; BA = Bahia; CE = Ceará; PB = Paraíba; PE = Pernambuco; SE = Sergipe; RJ = Rio de Janeiro; MG = Minas Gerais; SP = São Paulo; PR = Paraná; SC = Santa Catarina; MS = Mato Grosso do Sul; MT = Mato Grosso; DF = Distrito Federal.

### 2.2. Feline Hosts

Over the last twenty years, feline dirofilariosis has been primarily described in Brazil as a series of individual case reports (Figure 2). Only a limited number of large-scale studies have been undertaken, and, consequently, little is known about the prevalence of this parasite species. In recent studies, the prevalence of *D. immitis* among a study population of 556 cats from Rio de Janeiro city was 0.9% [24], while, a year later, the prevalence was 1.2% among 586 cats in the same region using the same diagnostic method (SNAP Feline Triple Test^®^, IDEXX Laboratories^®^) [54] (Table 2).

Although microfilaraemic cats are extremely rare [13], in 2020, researchers found microfilariae (mfs) in a FeLV-positive cat in Rio de Janeiro and later confirmed the diagnosis by serology and echocardiogram [54]. In 2018, a feline patient in respiratory distress was diagnosed with dirofilariosis using serological tests, and, after necropsy, this was confirmed by molecular testing of an adult worm found in the pulmonary artery [55]. This was the first time PCR was used to diagnose the condition in a cat. Later, in 2021, researchers used the same method to confirm the infection of a cat in northeastern Brazil [59].

Aberrant locations of adult *D. immitis* worms have been reported in feline patients, but these cases are particularly difficult to detect. An inguinal nodule with two adult worms identified as *Dioctophyme renale* and *Dirofilaria* spp. was described in a cat that was being neutered in Espírito Santo state in southeastern Brazil [58], with this being the first report of a parasite co-infection in the subcutaneous tissue.

### 2.3. Wildlife Hosts

There is a dearth of information on the prevalence of dirofilariosis in Brazilian wildlife. In 2006, Argentinian researchers defined the northeastern provinces of Argentina, which share borders with the South of Brazil, as high-risk areas for animal dirofilariosis [60], using four computer models based on temperature, occurrence, and number of mosquito species together with heartworm development units (HDU), a representation of the thermal energy required for larval development in the mosquito. However, only two studies have been performed in recent years in Iguaçu National Park, which also is an important tourist destination with thousands of visitors every year. In this area, which is shared between both countries, studies recorded prevalences of 1.48% [51] and 10.6% [5] for *D. immitis* and 33.2% [5] for *D. repens* in ring-tailed coatis (*Nasua nasua*), while no infection was detected in dogs [51] (Table 1 and Table 3). These results confirm the presence of *D. repens* in the Brazilian territory and raise the possibility that ring-tailed coatis could act as a reservoir of infection for both animals and humans living or visiting this area. Besides the epidemiological surveillance, over the last twenty years, only a single case has been reported in a Brazilian little spotted cat (*Leopardus tigrinus*) in the coastal city of Ubatuba, located in the state of São Paulo [61].

### 2.4. Human Hosts

Human dirofilariasis was first recorded in humans in Brazil in 1887 when Magalhães described a case in a child’s heart in Rio de Janeiro state [20]. Other reports have since followed, although there is reason to believe that the current prevalence of infection in the human population is underestimated. HPD patients are usually asymptomatic, and disease diagnosis is often incidental following chest radiography [62], when lesions may have been initially mistaken for a malignant nodular mass [63,64]. When present, clinical symptoms are non-specific and include fever, haemoptysis, cough, dyspnoea, and chest pain [8,65]. Definitive diagnosis is obtained following a pulmonary biopsy and histopathological examination, which is an invasive procedure [62] and unlikely to determine the actual species of *Dirofilaria* involved [66].

A small number of cases of human dirofilariasis have been reported in Brazil over the last twenty years (Table 4). The majority of HPD cases (*n = 7*) have been diagnosed in Florianopolis, Santa Catarina State, where canine dirofilariosis prevalence is known to be low (2.1%) [31]. Of these seven cases, three were asymptomatic, three showed respiratory symptoms, and one patient reported unspecified right arm pain [67].

In 2004 in Southeast Brazil, a 45-year-old female patient was diagnosed with HPD in Rio de Janeiro after the resection of a 4.0 × 3.5 cm lung mass. A worm was found following biopsy and described as *D. immitis*, although no molecular diagnosis was performed [70]. In the same region years later, a citizen of São Miguel do Araguaia, a city in Goiás State in the Midwest of Brazil, was diagnosed with HPD in Ribeirão Preto, São Paulo State, after a transthoracic lung biopsy. This was the first report of secondary myocarditis with dilated cardiomyopathy associated with the disease [71]. In addition to HPD, intraocular and oral presentations of dirofilariosis have also been described over the last twenty years [68,69]. The intraocular case was particularly important for two reasons: it was the only case report in which molecular diagnosis was implemented, and, surprisingly, a genomic variant of *D. immitis* was reported, as will be discussed below [68].

Finally, in a recent report of a 50-year-old female patient with HPD, researchers concluded by suggesting that vector control and dog immunisation are both important preventive measures to minimise the risk of infection in humans [62]. However, to date, vaccination against dirofilariosis in dogs is not yet available. The control of infection in dogs, with a view to preventing the spread of infection to humans, relies instead on the chemoprophylactic administration of macrocyclic lactones, the adequate use of diagnostic tools, and constant epidemiological surveillance. This underlines the importance of involving veterinarians as agents of public health in order to fully appreciate the epidemiological aspects of the diseases and to assist in rapid and effective diagnosis, treatment, and preventive measures.

## 3. Genomic Diversity of *Dirofilaria* in South America

*Dirofilaria immitis* has been detected in dogs in every region of Brazil, while *D. repens* has only been found in wildlife in the southern region. However, only in a proportion of cases has the identity of the parasite been definitively established since molecular diagnosis is rarely undertaken [18]. Notably, the molecular studies performed to date in Brazil have provided some evidence of genetic variation between Brazilian and Old World isolates [1,6,18,68,72]. For instance, in 2008, a 16-year-old boy was diagnosed with ocular dirofilariasis in Pará State, and the specimen, a male *Dirofilaria* spp. worm, was morphologically identified as *D. immitis*. However, when the parasite’s DNA was amplified and sequenced, considerable differences were found between it and eight GenBank *D. immitis* isolates from Australia and Italy. These equated to nucleotide identities of 94% and 95% for the *cox1* gene and 12 rDNA locus, respectively, reinforcing the idea that a different filarial species may be involved in the epidemiology of dirofilariosis in Brazil [68].

A number of findings support the contention that a genetically distinct form of *D. immitis* circulates in South America [12], which may be more virulent than its European counterparts. Brazilian researchers employed molecular techniques to amplify the 5.8S-ITS2-28S rDNA gene segments of adult specimens and blood samples from Ilha de Marajó (Pará State, Brazil) and compared the resulting sequences to those reported in other endemic areas available in GenBank [72]. In comparison to the reference sequence of *D. immitis* (Accession Number: AF217800), they found mutations in the 5.8S region (three, at positions 5, 16, and 26) and in the ITS2 region (four, at positions 68, 96, 152, and 258) not only in the isolate from Ilha de Marajó Island but also in a positive control sequence from an adult worm from Rio de Janeiro State, Brazil. This suggests that *D. immitis* in Brazil may be genetically distinct from the parasite in Europe [72].

Genomic diversity between *Dirofilaria* spp. isolates from Europe and other parts of the world is to be expected, and supporting evidence has been provided in recent studies [6,73,74]. Although insufficient proof was presented to define a new species of *Dirofilaria* [75], researchers from Hong Kong suggested that a novel species of the parasite, referred to as Candidatus *Dirofilaria hongkongensis,* is circulating in Brazil [73]. In their study, DNA sequences obtained from two genetic loci of three adult worms extracted from human lesions were compared and found to be identical to each other and identical to sequences found in blood samples of six local dogs. However, in comparison to sequences from existing Old World isolates, the *cox1* gene showed only 96.2% identity to *D. repens* and 89.3% to *D. immitis* [73]. Researchers from Sri Lanka subsequently reported a 36.9% prevalence of filarial infection in their country, with more than 80% of the sequences coinciding with *Dirofilaria* sp. *hongkongensis* [74]. Moreover, a subspecies of *D. repens*, or even a new *Dirofilaria* species, is suspected to be circulating in Chile on the basis of epidemiological surveillance combining parasitological and molecular tests first performed in the country only in 2012 [6]. A total of 102 mfs were recovered from 22 positive dogs and were subsequently morphologically analysed. Microfilaria from 18 dogs were found to have morphological similarities with *D. repens* but measured outside the normal range (360–410 µm in length; 7–8 µm in width). The mfs DNA extracted from three of these dogs was amplified by targeting the mitochondrial 12S rDNA gene, whose sequences showed only 95% identity with *D. repens* (Accession Number: AM779775).

In Argentina, the distribution of *Dirofilaria* spp. extends from the northern provinces southward to the city of La Plata (34°55′00″ S, 57°57′00″ W) [60]. However, in 2017, researchers reported eight cases of canine dirofilariosis in the province of Neuquén, in Patagonia (38°57′06″ S 68°04′28″ W), with notable findings from a morphological, antigenic, and molecular point of view [76]. The length of the mfs was found to be 370 µm on average. This is much longer than has been described for *D. immitis* to date, which is also the only species known to be circulating in the country [76]. Surprisingly, the *D. immitis* antigen detection test (FASTest^®^ DIRO, Megacor, Austria) was negative in all cases. DNA was amplified and sequenced for the 5.8S-ITS2-28S and COI loci for five and two individuals, respectively. All of these showed less than 95% sequence identity with other filarial species deposited at GenBank, including *D. repens* from Tunisia and the Czech Republic, *D. immitis* from Portugal and Spain, and *Dirofilaria* sp. *hongkongensis*, suggesting that a hitherto unknown species may be circulating in Argentina [76].

These studies suggest that novel species and/or subspecies of *Dirofilaria* may exist, however further studies are required in this area [1,6,18,68,72]. Whether the atypical *Dirofilaria* strains detected in Brazil and the neighbouring countries of Chile and Argentina are novel species or simply geographical variants of recognised species is yet to be determined. Genomic analyses, employing next-generation sequencing, may be used to accurately characterise filarial populations and identify species in circulation. For example, recent studies using long-read metabarcoding (MinIon™, Oxford Nanopore Technologies) have enabled the complete COI gene sequence of *D. immitis* to be determined. This technology may be applied to monitoring the spread of known pathogens and detecting emerging species or subspecies, particularly in areas where more than one species is present [77]. Thus, the issue of variant *Dirofilaria* strains circulating in Brazil may be investigated and clarified using these tools.

## 4. Treatment and Prophylaxis Available in Brazil

Melarsomine, the preferred adulticide drug for treating canine dirofilariosis, is unavailable in many countries including Brazil [78]. In such areas, “slow-killing” strategies, consisting of a combination of macrocyclic lactones at chemoprophylaxis dosages together with doxycycline, are the only alternative to treat canine patients [79,80,81]. Currently, the most popular treatment protocol in Brazil consists of the monthly application of imidacloprid 10% and moxidectin 2.5% (Advocate^®^ 4–10 mg/kg), together with doxycycline at a dose of 10 mg/kg once or twice a day, for the first 30 days [43,78]. Although the application of melarsomine could resolve infection within six months depending on the severity of the infection and the dog’s health status, the combination of imidacloprid and moxidectin takes between 12 to 24 months to clear infection [80].

The treatment of dirofilariosis is also a challenge in cats, as adulticides such as melarsomine are toxic and only macrocyclic lactones can be used to reduce microfilaraemia [24,54]. In order to reduce the prevalence of feline dirofilariosis, prevention combined with continuous epidemiological surveillance and follow-up of positive cases is essential, and this may entail symptomatic treatment, immunological testing, laboratory testing, radiography, and echocardiography [56,57,82].

The prevention of dirofilariosis is crucial in endemic areas. The American Heartworm Society (AHS) recommends year-round administration of macrocyclic lactones associated with vector control, and, although this information is widely available for the international research community, there is an information gap in the websites known to be most commonly accessed by small animal practitioners in Brazil. To the best of our knowledge, the only Brazilian website with information regarding chemoprophylaxis belongs to the Instituto de Medicina Veterinária do Coletivo (IMVC). In this, they summarise and translate the AHS guidelines but do not provide any information about the chemoprophylactic drugs available in Brazil. Similarly, the Federal Council of Veterinary Medicine (CFMV), the organisation that regulates veterinary practice throughout Brazil, does not provide any technical information on this topic.

In the city of Rio de Janeiro, where the prevalence of dirofilariosis is 7.0% and 0.9% in dogs and cats, respectively [24], only 174/498 dogs (34.9%) and 18/107 cats (16.8%) received at least one dose of macrocyclic lactones in 2023 (unpublished data). With melarsomine being unavailable in Brazil, the need for a year-round prophylactic scheme for controlling the disease and preventing deaths is abundantly clear [24].

## 5. Discussion

This review summarised, for the first time, all *Dirofilaria* spp. reports and prevalence studies conducted in the last decade among dogs and in the last twenty years in feline, wildlife, and human populations in Brazil. Dogs are the definitive host and a key sentinel species for this parasite [13] and based on canine *D. immitis* occurrence, the highest prevalence was recorded in the interior of the country (Figure 2), in particular in the northern [26,27], northeastern [31,38,40,42] and southeastern [23,31,32,46,47] cities. It is interesting to note that these municipalities are inland, close to major river systems, thus confirming the occurrence of the parasite in dogs in the non-coastal regions of the country [48]. Although there is some awareness among veterinarians and clinicians regarding the parasite’s presence in coastal areas, in the interior of the country, it is not recognised nearly to the same extent. In fact, in Porto Velho, Rondônia state in the Brazilian hinterland, the first report of *D. immitis* prevalence in dogs (12.8%) was only published in 2013 [36]. This new-found knowledge indicates the need for the implementation of monitoring and surveillance activities, especially in those areas near the Amazonas state. This area is constantly under pressure from forest devastation for timber extraction, fires, and agribusiness, and thus, there is a substantial ongoing impact on the Amazon biome and its ecosystem [83]. It may be hypothesised that these activities may also exert an influence on both parasite and mosquito populations.

In the southeastern region, Rio de Janeiro state has the highest number of published prevalence studies of dirofilariosis in Brazil [18,23,24,31,32,44,45,46,47,48,49,54]. Similar to the northern regions, the southern part of the country is also experiencing ongoing environmental challenges, such as flooding and the effects of climate change, alongside substantial socioeconomic issues. There have been several prevalence studies of dirofilariosis in dogs in this area, with a few prevalence reports and cases of *D. immitis* in cats recorded but only a single human case reported. The state capital has a tropical climate and over six million inhabitants, the majority of which live in deprivation. There exist serious problems of environmental pollution, with unprocessed sewage being routinely released in streams or rivers. Thus, due to cost, there is little access to chemoprophylaxis and the prevailing environmental factors may promote a flourishing mosquito vector population [63,84,85]. *Dirofilaria immitis* prevalence among dogs from the city varied from 5.8 to 20% (Table 1). The outskirts of Rio de Janeiro are also very populated municipalities, such as Niteroi, with a very high parasite prevalence ranging from 24.1 to 58.6% in dogs [18,31]. Additionally, in the coastal area of the state, also known as the Lake Region, 100 km from the state capital, in four municipalities (Cabo Frio, Armação de Búzios, São Pedro da Aldeia, and Araruama) the prevalence of dirofilariosis in dogs is the highest of the whole country, ranging from 15.5 to 62.2% [23,31,32,47]. These high prevalences can be partially explained by the environmental conditions of this region, which is rich in lakes, lagoons, sea, and a vast rural area that may favour mosquito vector development [13]. In addition, its permanent population of 644,332 inhabitants [86] doubles with the year-round tourist business, that increases housing demand, leads to the occupation of natural protected areas and worsens the already limited sewage system [87]. The combination of natural conditions and ongoing human activities puts the region at high risk of flooding [87].

In the southern region of the country, the prevalence in dogs varied from 0.4 to 31.8% in the coastal areas of Paraná and Santa Catarina states, while in the interior, at the Iguaçu National Park, Iguaçu River Waterfalls, the prevalence in dogs varied from 16 to 22% [5]. This is the only location in the country where the parasite has been detected in wild animals (i.e., ring-tailed coatis) [5,51], underlining the potential role of wildlife as a novel source of *Dirofilaria* spp. infection for humans, alongside the canine host. Further studies should be conducted to elucidate the impact of wildlife on the epidemiology of the disease as well as their potential role in harbouring other zoonotic filarial worms such as *Dirofilaria tenuis*, *Dirofilaria ursi* and *Dirofilaria magnilarvata*, and especially *Dirofilaria striata* and *Dirofilaria spectans*, which have already been described in Brazil [88]. Epidemiological surveys in unexplored Brazilian regions can help us solve the mystery of how *D. repens* arrived in Foz de Iguaçu, about 1800 km northeast of the metropolitan area of Santiago, Chile [6], the only report of this species in South America until 2017.

On the island of Florianópolis, the coastal capital of Santa Catarina state, the majority of human reports have been recorded in the last twenty years [67]. Remarkably, the prevalence of infection in dogs in Florianópolis has decreased by almost 10% over twelve years, which has been attributed to urbanisation and human population growth [31], and it is possible that a similar drop will be also seen for the human cases.

The varying patterns in *D. immitis* distribution in Brazil may be related to the impact of environmental changes and vulnerability, human migration, poorly organised urbanisation, social deprivation, and vector ecology. The changing climate continues to alter environmental conditions, which in turn influences the dynamics and the evolving epidemiology of dirofilariosis in Brazil; outbreaks of infectious diseases in Brazil have been variously linked to climatic change, including El Niño, La Niña, heat waves, droughts, floods, rising temperatures, and increased rainfall [89,90]. These factors could potentially expose new regions to the risk of disease [83,89], thus this mosquito-borne parasitic condition should be considered as a neglected disease in some regions of Brazil, and an investment in broad-ranging and ongoing research is urgently needed. As there exists preliminary evidence that a novel *Dirofilaria* species or subspecies may be circulating in the Americas, studying the molecular epidemiology of the parasite in Brazil is also of great importance. This could provide valuable insight into the efficacy of diagnostic tools, treatment, and the design of control and prevention strategies, which are currently based primarily on an understanding of European parasites. Genomic studies offer the possibility of investigating the evolutionary history of the parasite and, potentially, its local adaptation and interaction with host species.

Long-term epidemiological surveillance is essential for monitoring and identifying emerging disease hotspots to safeguard the health of both humans and animals. In addition to general surveillance monitoring, in endemic areas, dogs should be tested ideally every six months and receive monthly doses of preventive treatment with macrocyclic lactones [91]. Additionally, veterinarians should be aware of the prevalence of infection in their region and the strengths and limitations of each diagnostic tool available. In summary, it is essential that this disease is considered from a One Health standpoint, in which the importance of the interconnection between human, animal, and environmental health is appreciated, enabling clinicians and veterinarians to work together to control this important zoonotic disease [84,85,92].

## 6. Conclusions

The varying prevalence of *Dirofilaria* spp. across the Brazilian regions together with evident knowledge gaps on the impact of the disease on feline, human, and wildlife populations underscore the complexity of controlling this neglected disease and emphasises the need for ongoing epidemiological surveillance and research. Genomic studies designed to investigate the circulation of putatively novel species or subspecies in the Americas are crucial for developing a suite of effective diagnostic, treatment, and prevention strategies tailored to Brazil’s unique ecological and socioeconomic status.

## Figures and Tables

**Table 2:** Feline dirofilariosis prevalence and case reports over the last twenty years (2004–2024) in Brazil, organised by region, city, and state, and the different diagnostic methods employed.

Region	State	City	Filarial Species	Prevalence	Positives/Total	Diagnostic Method	Authors
Southeast	RJ	Rio de Janeiro	*D. immitis*	0.9%	5/556	SNAP Feline Triple Test (IDEXX Laboratories, Westbrook, ME, USA)	[24]
	RJ	Rio de Janeiro	*D. immitis*	1.2%	7/586	SNAP Feline Triple Test (IDEXX Laboratories, Westbrook, ME, USA)	[54]
	RJ	Niterói	*D. immitis*	Case report	1/1	PCR (12S and cox1)	[55]
	RJ	Ilha do Governador	*D. immitis*	Case report	1/1	Blood smear, Knott’s method, SNAP Feline Triple Test (IDEXX Laboratories, Westbrook, ME, USA) and echocardiogram	[56]
	RJ	Rio de Janeiro	*D. immitis*	Case report	1/1	Clinical signs, radiography, and necropsy	[57]
	ES	Alegre	*D. immitis*	Case report	1/1	Morphological	[58]
Northeast	RN	Mossoró	*D. immitis*	Case report	1/1	PCR (18s, MyoHC and hsp70)	[59]

RJ = Rio de Janeiro; RN = Rio Grande do Norte.

**Table 3:** Wild animals’ *Dirofilaria* spp. prevalence and case reports over the last twenty years (2004–2024) in Brazil, organised by region, state, and city and the different diagnostic methods employed.

Region	State	City	Animal	Filarial Species	Prevalence	Positives/Total	Diagnostic Method	Authors
South	PR	Parque Nacional Iguaçu	Ring-tailed coatis (*Nasua nasua*)	*D. immitis*	10.7%	8/75	Knott’s method, Histochemical, Serology (Witness Dirofilaria, Zoetis®, Parsippany, NJ, USA)	[5]
	PR	Parque Nacional Iguaçu	Ring-tailed coatis (*Nasua nasua*)	*D. repens*	33.3%	25/75	Knott’s method, Histochemical	[5]
	PR	Parque Nacional Iguaçu	Ring-tailed coatis (*Nasua nasua*)	*D. immitis*	1.48%	2/135	Knott’s method, Immunochromatography and PCR (myoHC and hsp70)	[51]
Southeast	SP	Ubatuba	Brazilian little spotted cat (*Leopardus tigrinus*)	*D. immitis*	Case report	1/1	Clinical signs, radiography, and necropsy	[61]

PR = Paraná; SP = São Paulo.

**Table 4:** Human cases of dirofilariasis due to *Dirofilaria immitis* recorded in the last twenty years in Brazil, organised by city, state, and lesion location.

Region	State	City	Patient	Symptoms	Location	Authors
North	PA	Belém	M, 16 yo	Yes	Eye	[68]
Northeast	PB	João Pessoa	F, 65 yo	Yes	Oral cavity	[69]
Southeast	RJ	Rio de Janeiro	F, 45 yo	Yes	Lung	[70]
	SP	Ribeirão Preto *	M, 67 yo	Yes	Lung	[71]
South	PR	Curitiba	F, 50 yo	Yes	Lung	[62]
	SC	Florianópolis	M, 75 yo	No	Lung	[67]
	SC	Florianópolis	M, 42 yo	No	Lung	[67]
	SC	Florianópolis	M, 70 yo	Yes	Lung	[67]
	SC	Florianópolis	M, 55 yo	Yes	Lung	[67]
	SC	Florianópolis	M, 44 yo	Yes	Lung	[67]
	SC	Florianópolis	F, 69 yo	Yes	Lung	[67]
	SC	Florianópolis	F, 63 yo	Yes	Lung	[67]

PA = Pará; PB = Paraíba; RJ = Rio de Janeiro; SP = São Paulo; PR = Paraná; SC = Santa Catarina; M = male; F = female; yo = Years-old. * Living in São Miguel do Araguaia—GO (Midwest of Brazil).

## Data Availability

No new data were created or analysed in this study. Data sharing is not applicable to this article.
